# Proteinase-activated receptor 2 promotes TGF-β-dependent cell motility in pancreatic cancer cells by sustaining expression of the TGF-β type I receptor ALK5

**DOI:** 10.18632/oncotarget.9600

**Published:** 2016-05-27

**Authors:** Franziska Zeeh, David Witte, Thomas Gädeken, Bernhard H. Rauch, Evelin Grage-Griebenow, Nadja Leinung, Sofie Joline Fromm, Stephanie Stölting, Koichiro Mihara, Roland Kaufmann, Utz Settmacher, Hendrik Lehnert, Morley D. Hollenberg, Hendrik Ungefroren

**Affiliations:** ^1^ First Department of Medicine, University of Lübeck, Lübeck, Germany; ^2^ Department of General Pharmacology, Institute of Pharmacology, University Medicine Greifswald, Greifswald, Germany; ^3^ Department of Physiology & Pharmacology and Department of Medicine, Inflammation Research Network-Snyder Institute for Chronic Diseases, University of Calgary, Cumming School of Medicine, Calgary, AB, Canada; ^4^ Department of General, Visceral and Vascular Surgery, Jena University Hospital, Jena, Germany

**Keywords:** TGF-β, PAR2, ALK5, signalling, cell motility

## Abstract

Pancreatic ductal adenocarcinoma (PDAC) is characterized by high expression of transforming growth factor (TGF)-β and the G protein-coupled receptor proteinase-activated receptor 2 (PAR2), the latter of which functions as a cell-surface sensor for serine proteinases asscociated with the tumour microenvironment. Since TGF-β and PAR2 affect tumourigenesis by regulating migration, invasion and metastasis, we hypothesized that there is signalling crosstalk between them. Depleting PDAC and non-PDAC cells of PAR2 by RNA interference strongly decreased TGF-β1-induced activation of Smad2/3 and p38 mitogen-activated protein kinase, Smad dependent transcriptional activity, expression of invasion associated genes, and cell migration/invasion *in vitro*. Likewise, the plasminogen activator-inhibitor 1 gene in primary cultures of aortic smooth muscle cells from PAR2^−/−^ mice displayed a greatly attenuated sensitivity to TGF-β1 stimulation. PAR2 depletion in PDAC cells resulted in reduced protein and mRNA levels of the TGF-β type I receptor activin receptor-like kinase 5 (ALK5). Forced expression of wild-type ALK5 or a kinase-active ALK5 mutant, but not a kinase-active but Smad-binding defective ALK5 mutant, was able to rescue TGF-β1-induced Smad3 activation, Smad dependent transcription, and cell migration in PAR2-depleted cells. Together, our data show that PAR2 is crucial for TGF-β1-induced cell motility by its ability to sustain expression of ALK5. Therapeutically targeting PAR2 may thus be a promising approach in preventing TGF-β-dependent driven metastatic dissemination in PDAC and possibly other stroma-rich tumour types.

## INTRODUCTION

Pancreatic ductal adenocarcinoma (PDAC) is a highly malignant tumour with a still dismal prognosis. In Western countries, PDAC ranks 4^th^ in the order of death-related tumour diseases with a prevalence that is still increasing [1]. PDAC is normally detected at an advanced stage when the patients present with metastases. As a consequence, therapeutic options are very limited leading to a very low overall 5-year survival rate of less than 5% [2]. PDAC is characterized by overexpression of transforming growth factor-β (TGF-β). Overexpression of this growth factor is associated with more aggressive disease and shorter survival [3] and this can be explained by the ability of TGF-β1 to enhance epithelial-to-mesenchymal transition (EMT), angiogenesis, migration, invasion, and metastasis [4–7]. The importance of TGF-β signalling in pancreatic cancer is emphasised by the finding that the TGF-β signal transduction pathway is one of only four cellular signalling pathways that are genetically altered in 100% of pancreatic tumours [8].

TGF-β signals through two highly glycosylated, membrane-bound receptors designated type II (TβRII) and type I/activin receptor-like kinase 5 (ALK5). After being phosphorylated by TβRII on serine/threonine residues, ALK5 activates the canonical Smad pathway and eventually also triggers non-Smad pathways, *e.g.* p38 mitogen-activated protein kinase (MAPK) signalling [9]. Phosphorylation of the receptor-regulated Smads, Smad2 and Smad3, at their C-terminal end by the ALK5 kinase represents a crucial step in regulating TGF-β signalling. Activated receptor regulated Smads (R-Smads) subsequently form a complex with Smad4 which moves into the nucleus to regulate the transcriptional activity of TGF-β sensitive target genes [4–7, 9].

Proteinase-activated receptors (PARs) represent a subgroup of G protein-coupled receptors [10, 11] that currently comprise four members (PAR1-4). PARs exhibit a unique mechanism of proteolytic activation. Serine proteinases are able to cleave the receptor at specific recognition sites within the extracellular N-terminus leading to the exposure of amino-terminal “tethered ligand” sequences that remain attached to the receptor and bind to the extracellular receptor domains to trigger conformational changes and various signalling events such as activation of G proteins, the β-arrestin pathway and transactivation of a variety of receptors and other signalling molecules [11, 12]. The principal enzyme activators for PAR2 are trypsin and activated factor X (FXa) both of which cleave PAR2 at its ‘canonical’ R//S tethered ligand-generating activation site [10–12]. PAR2 (encoded by *F2RL1*) is highly expressed in pancreas, where it functions as a key molecule in regulation of pancreatic exocrine secretion [13]. However, PAR2 has also been shown to drive tumour growth in murine models of mammary adenocarcinoma [14] and pancreatic cancer. Silencing *F2RL1* in a PDAC cell line by RNA interference or genetically ablating it from the stromal compartment dramatically suppressed the growth of subcutaneous tumour xenografts and of orthotopically growing primary tumours, respectively [15, 16].

PDAC tissue is characterized by a desmoplasia, a well-developed stromal compartment consisting of fibroblasts, endothelial cells, immune cells, soluble (hormones, growth factors) and non-soluble (extracellular matrix) molecules. Within this highly complex tumour microenvironment both the cancer cells and the stromal cells coexpress TβRII, ALK5, and PAR2 [17] and secrete large amounts of TGF-β and potential PAR2 ligands. TGF-β1 and PAR2 can mutually upregulate their expression and both can induce other profibrogenic genes [17, 18], contributing to the desmoplastic reaction in pancreatic cancer [19]. Since a fibrotic and proinflammatory environment is known to favor metastatic dissemination, it is not surprising that both TGF-β /ALK5 [4–7] and PAR2 [19–23] have been shown to promote cell motility, invasion and metastasis formation across a large variety of cancers including PDAC.

PAR2 can cooperate with PAR1 and various other types of receptors [12], but whether both PARs also interact with the TGF-β receptor(s) has remained unclear. Burch and coworkers were the first to describe PAR1 transactivation of ALK5 in the regulation of thrombin-induced proteoglycan synthesis in vascular smooth muscle cells [24, 25]. More recently, we observed that PAR2 transactivation of ALK5 and epidermal growth factor receptor signalling pathways can contribute to renal fibrosis [26]. However, whether, in turn, PAR2 is required for TGF-β /ALK5 signalling and, if so, whether this impacts TGF-β responses is not known. Given PAR2 and TGF-β colocalization in PDAC tissue, the overlapping spectra of cellular activities and the mutual regulatory interactions, we hypothesized that there is signalling crosstalk between PAR2 and TGF-β in tumour cells to promote TGF-β pro-oncogenic effects and PDAC progression. To study this, we employed cell lines of PDAC and non-PDAC origin with well characterized TGF-β1 sensitivity and expression/function of PAR2 [15, 27, 28].

## RESULTS

### Depletion of PAR2 protein suppresses TGF-β1-induced migration and invasion

**Figure 1:**
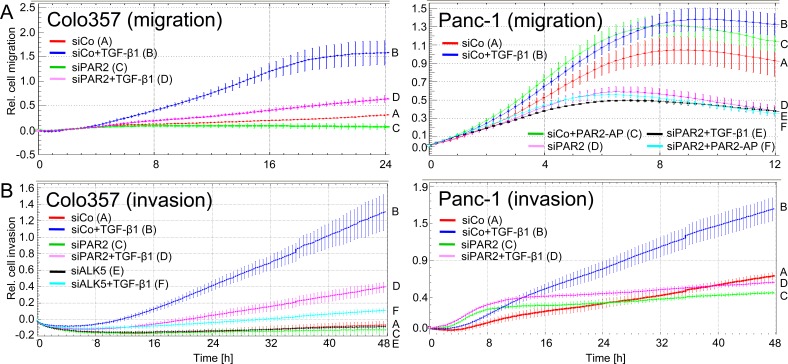
RNA interference mediated cellular depletion of PAR2 suppresses TGF-β1-induced cell motility The coloured curves indicate the real-time cell migration data of the various cell populations with the slope of the curve being proportional to the migration velocity of cells. **A.** Cell migration in Colo357 and Panc-1 cells transiently transfected with scrambled control siRNA (siCo) or PAR2 siRNA (siPAR2) and treated or not with TGF-β1 during the entire assay. For Colo357 cells, differences between siCo + TGF-β1 (left-hand graph, blue curve/B) and siPAR2 + TGF-β1 (left-hand graph, magenta curve/D) are significant at the 8 h and all later time points. For Panc-1 cells, data for siCo + TGF-β1 (left-hand graph, blue curve/B) and siPAR2 + TGF-β1 (right-hand graph, black curve/E), and between siCo + PAR2-AP (right-hand graph, green curve/C) and siPAR2 + PAR2-AP (right-hand graph, cyan curve/F) are significantly different at the 8 h and all later time points. **B.** The same as in A except that the assay was run in an invasion setup with Matrigel as barrier. In addition, ALK5 siRNA was used as a control for blocking the pro-invasive TGF- effect (for validation of this siRNA in Colo357 cells see Figure 5B). Data for siCo + TGF-β1 (blue curve/B) and siPAR2 + TGF-β1 (magenta curve/D) from both cell lines are significantly different (*p* < 0.05) at the 16 h and all later time points. Data in A and B were derived from three parallel wells, represent the mean ± SD and are representative of three assays. Letters on each graph's right-hand side allow for colour independent identification of the curves.

Both PAR2 and TGF-β have been implicated in the control of cell motility. To analyse whether PAR2 expression is crucial for TGF-β1-induced cell migration and invasion, we depleted various PDAC and non-PDAC cell lines of PAR2 by transient transfection of siRNA (a pool of three prevalidated Stealth siRNAs) and subjected them to the xCELLigence^®^ RTCA migration assay. Due to the inability of all available PAR2 antibodies including the clone SAM11 from Santa Cruz Biotechnology to recognize endogenous PAR2 in immunoblots [Refs. 29, 30, and our own unpublished results], we employed quantitative real-time RT-PCR (qPCR) analysis to demonstrate reduced total PAR2 expression (Supplementary Figure 1A) and flow cytometry to verify a concomitant decrease in cell surface associated PAR2 expression (Supplementary Figure 1B) in response to siRNA transfection. Interestingly, the ability of TGF-β1 to stimulate migration in PAR2 knockdown transfectants was greatly reduced or abolished in Colo357 and Panc-1 cells (Figure 1A), IMIM-PC1 (data not shown) and HaCaT cells (Supplementary Figure 2). As a further control for specificity of the PAR2 siRNA effect, Panc-1 cells depleted of PAR2 were treated with the PAR2 selective agonistic peptide, SLIGKV-NH_2_ (PAR2-AP). As expected, migratory activity afforded by PAR2-AP was completely lost (Figure 1A, right-hand graph). Another set of experiments was then performed using an invasion mode of the RTCA assay with Matrigel as a barrier. Similar to ALK5 siRNA, as positive control for blunting any TGF-β1 signalling, siRNA to PAR2 blocked TGF-β1-induced cell invasion in both Colo357 and Panc-1 cells (Figure 1B). In summary, these data clearly show that PAR2 expression is crucial for TGF-β1-dependent cell motility *in vitro*.

### Depletion of PAR2 inhibits expression of TGF-β target genes involved in cell invasion

**Figure 2:**
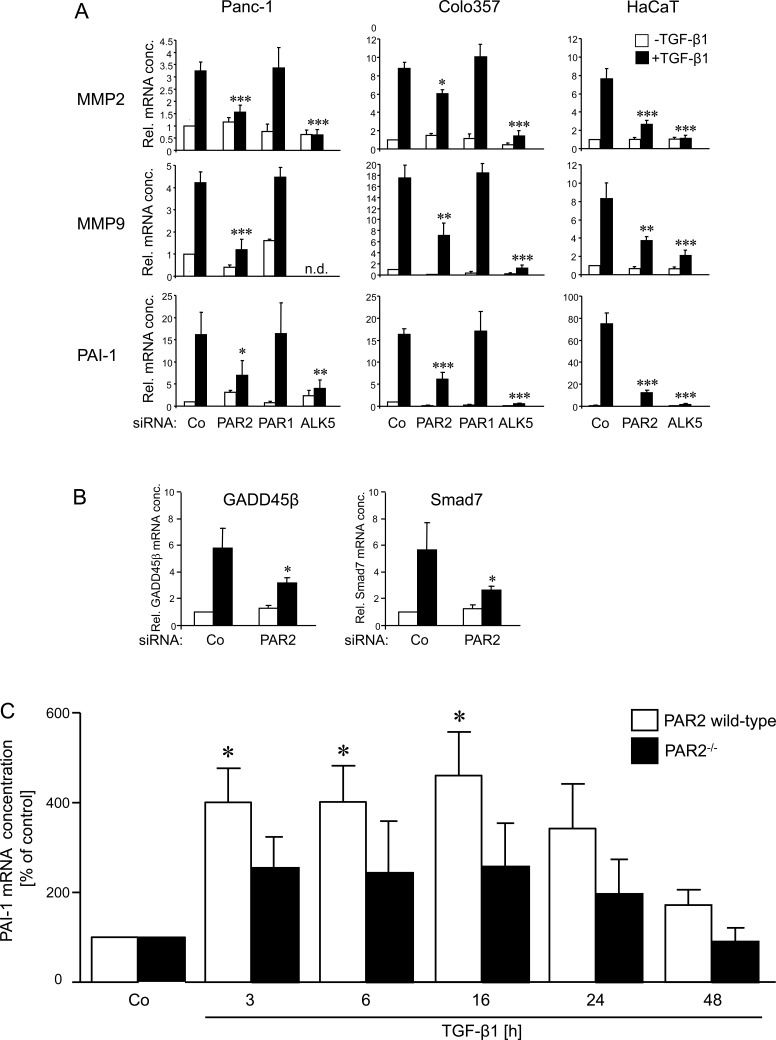
Effects of PAR2 depletion on the TGF-β1 response of invasion associated genes **A.** Panc-1, Colo357, and HaCaT cells were transfected twice on two consecutive days with 50 nM of control (Co), PAR2, PAR1 or ALK5 siRNA, as indicated, using Lipofectamine RNAiMAX. Twenty-four h after the second round of transfection, cells were stimulated, or not, with 5 ng/ml TGF-β1 for another 24 h (Panc-1, Colo357) or 48 h (HaCaT) followed by RNA isolation and qPCR for the indicated genes. **B.** Panc-1 cells were transfected as described in A using the indicated siRNAs. Twenty-four h after the second round of transfection, cells were stimulated, or not, with 5 ng/ml TGF-β1 for 1 h followed by RNA isolation and qPCR for GADD45β and Smad7. In A and B, bars represent mean values ± SD of three wells normalised to β-actin and TBP. Successful knockdown of PAR2, ALK5 and PAR1 expression was verified by qPCR (not shown). One representative experiment out of three experiments performed in total is shown. *P* < 0.05 relative to the respective TGF-β1-treated Co. **C.** Primary aortic smooth muscle cells were isolated from PAR2^−/−^ and strain-matched (C57/Bl6J) control wild-type mice and cultured in DMEM with 10% FBS. Prior to stimulation, the aortic smooth muscle cells were seeded in 6-well plates at about 70% confluence. After 24 h of serum withdrawal, cells were incubated with 5 ng/ml TGF-β1 for the times indicated and subsequently processed for RNA isolation and qPCR analysis for PAI-1 and GAPDH. Data shown represent mean values ± SD after normalisation for GAPDH from eight mice per group. *P < 0.05 relative to the untreated Co set arbitrarily at 100. Note that in PAR2^−/−^ mice none of the mean values from TGF-β1-treated cells were significantly different from Co.

Prompted by the data in Figure 1, we hypothesised that PAR2 will also affect genes involved in cell migration and invasion in such a way that their TGF-β response is lost or reduced. To test this prediction, PAR2 siRNA or PAR1 siRNA transfected Panc-1 and Colo357 cells, and PAR2 siRNA transfected HaCaT cells were treated with TGF-β1 and analysed by qPCR for expression of the TGF-β -responsive genes matrix metalloproteinase 2 (MMP2), MMP9 and plasminogen activator inhibitor-1 (PAI-1) [7]. As predicted, depletion of PAR2 but not PAR1 led to either a loss or at least a strong decrease in the responsiveness of the tested genes to TGF-β1 stimulation in all three cell lines (Figure 2A). PAR2 depletion in Panc-1 cells also resulted to a failure of TGF-β1 to induce the expression of factors involved in positive (GADD45b [31, 32]) and negative (Smad7 [33]) feedback regulation of Smad signalling (Figure 2B). These results provide another example of the importance of PAR2 expression for proper regulation of a wide variety of individual TGF-β target genes and provide a molecular explanation for the crucial role of PAR2 in TGF-β -induced migration and invasion (see Figure 1).

PAI-1 is an established and sensitive TGF-β target gene across diverse species and across cells of different developmental origin. It is the major physiologic inhibitor of the plasmin-based pericellular cascade and a causative factor in the development of vascular thrombotic and fibroproliferative disorders [34]. The availability of mice in which *F2RL1* had been ablated (PAR2^−/−^ mice) prompted us to study whether PAR2 also affects TGF-β signalling in primary non-transformed cells of mesodermal origin, such as smooth muscle cells. To do this, smooth muscle cells were isolated from the aorta of PAR2^−/−^ and strain-matched (C57/Bl6J) wild-type mice, stimulated in culture for various times with TGF-β1 and analysed for expression of PAI-1. As shown in Figure 2C, TGF-β1-induced PAI-1 expression was significantly reduced in PAR2^−/−^ compared to wild-type mice. These data suggest the possibility that the PAR2-TGF-β crosstalk is also operating in non-neoplastic cells of murine origin and is attenuated in PAR2 deficient mice, at least with respect to TGF-β1-dependent PAI-1 expression.

### Modulation of PAR2 expression alters TGF-β1/Smad dependent reporter gene activity

**Figure 3:**
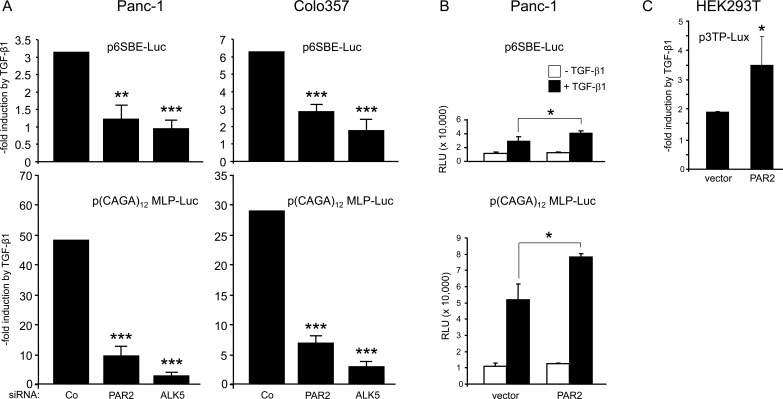
Depletion of PAR2 decreases the sensitivity of TGF-β/Smad responsive reporters to TGF-β1 stimulation **A.** Panc-1 and Colo357 cells were transfected on day 1 with RNAiMAX along with negative control siRNA (Co), PAR2 siRNA or ALK5 siRNA. On day 2, cells received the same siRNAs along with either p6SBE-Luc (*upper* two graphs) or p(CAGA)_12_ MLP-Luc (*lower* two graphs), and the *Renilla* luciferase encoding vector pRL-TK-Luc using Lipofectamine 2000. Forty-eight h after the start of the first transfection, cells were stimulated with TGF-β1 for another 24 h followed by dual luciferase measurements. Data are the mean ± SD from six parallel wells. Asterisks indicate significance *vs.* TGF-β1-treated Co. **B.**, **C.** Ectopic expression of PAR2 cells increases the sensitivity of Smad responsive reporter genes to TGF-β1. **B**. Panc-1 cells were transiently transfected with either empty pcDNA3 vector (vector) or PAR2 encoding vector (PAR2-HA) along with either p6SBE-Luc (*upper* graph) or p(CAGA)_12_ MLP-Luc (*lower* graph), and pRL-TK-Luc. Two days later, cells were treated with 5 ng/ml TGF-β1 for 24 h followed by lysis and dual luciferase assay. Data represent the normalised mean ± SD of six wells. **C**. HEK293T cells were cotransfected with p3TP-Lux, pRL-TK-Luc, and either empty vector or PAR2 encoding vector. Forty-eight h after the start of transfection, cells were stimulated with TGF-β1 for another 24 h followed by dual luciferase measurements. Data represent the mean ± SD from six wells. Data in A-C are representative of at least four independent experiments.

The above data indicated that PAR2 protein expression was required for various TGF-β1- induced responses that are known to be Smad dependent. To test in a more direct fashion whether PAR2 is required for TGF-β1/Smad-dependent transcription, cells were transiently transfected with siRNA to downregulate either PAR2, or ALK5 as control, along with the Smad responsive reporter plasmids, p6SBE-Luc, p(CAGA)_12_ MLP-Luc, or p3TP-Lux. *F2RL1* silencing in Panc-1 and Colo357 cells strongly suppressed TGF-β1 induction of p6SBE-Luc and p(CAGA)_12_ MLP-Luc, although the inhibitory effect of silencing of *F2RL1* was not as strong as that of ALK5 (Figure 3A). Similar albeit less dramatic effects were seen for p6SBE-Luc in IMIM-PC1 cells and for p(CAGA)_12_ MLP-Luc in HaCaT cells (data not shown). Thus, siRNA-mediated downregulation of either PAR2 or ALK5 attenuated the induction of reporter gene activity by TGF-β1. Given the lower reporter gene activity in PAR2 depleted cells, we asked whether, conversely, ectopic overexpression of PAR2 would enhance it. Upon cotransfection of a PAR2 expression vector and p6SBE-Luc, or p(CAGA)_12_ MLP-Luc, Panc-1 cells responded to a 24-h TGF-β1 treatment with a clear increase in luciferase activity over that of empty vector transfected control cells (Figure 3B). Very similar results were obtained in HEK293T cells cotransfected with PAR2 and p3TP-Lux (Figure 3C). These data show that increasing the cellular levels of PAR2 can enhance the sensitivity to TGF-β1/Smad mediated transcription.

### Depletion of PAR2 inhibits TGF-β1-induced phosphorylation of Smad3, Smad2 and p38 MAPK

**Figure 4:**
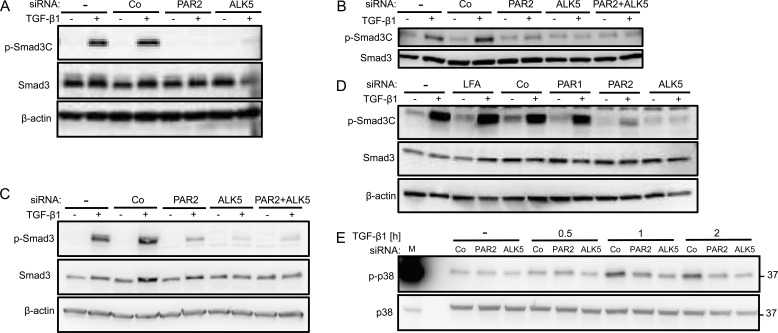
Specific inhibition of TGF-β1 mediated Smad3C phosphorylation following RNA interference mediated depletion of PAR2 **A.**-**E.** Immunoblot analysis of C-terminally phosphorylated Smad3 (p-Smad3C) in Panc-1 **A**. Colo357 **B**. IMIM-PC1 **C**. and HaCaT **D**. cells treated with transfection agent alone (−) or transiently transfected with 50 nM of control (Co) siRNA, or siRNA specific to PAR2, PAR1, or ALK5 as indicated. Forty-eight h after the start of transfection, cells were stimulated or not with TGF-β1 for 1 h. In A-D, blots were stripped and reprobed with antibodies to Smad3 and β-actin to control for equal loading. **E**. Immunoblot analysis of phospho-p38 (p-p38), and p38 as loading control, in Panc-1 cells transfected with the indicated siRNAs and treated or not (−) with TGF-β1 for various times as indicated. In each panel, a representative blot from at least three independent experiments is shown. Successful knockdown of PAR2, ALK5 and PAR1 expression was verified by qPCR (not shown). M, molecular weight marker.

Results so far have provided ample evidence that depletion of PAR2 abolished TGF-β /Smad signalling and Smad-dependent responses. To analyse whether this is also seen at the level of Smad activation, we first silenced *F2RL1* by siRNA in various PDAC and non-PDAC cell lines and subsequently performed immunoblotting with antibodies recognizing C-terminally phosphorylated Smad3 (p-Smad3C). Interestingly, the absence of PAR2 expression resulted in a consistent failure of Panc-1 (Figure 4A), Colo357 (Figure 4B), IMIM-PC1 (Figure 4C), and HaCaT cells (Figure 4D) to fully phosphorylate Smad3C in response to TGF-β1 stimulation. PAR2 siRNA also strongly reduced TGF-β1 induction of p-Smad2C in all three PDAC cell lines (Supplementary Figure 3). The inhibitory effect on Smad3C phosphorylation appears to be specific to PAR2 siRNA since parallel transfection of PAR1 siRNA in HaCaT cells failed to mimic this effect (Figure 4D).

Next we wanted to know whether PAR2 depletion also affected non-Smad signalling pathways involved in the induction of migration/invasion, such as the MTK1/TAK1-MKK3/6-p38 MAPK pathway. Since we and others have shown earlier that Panc-1 cells activate p38 MAPK in response to TGF-β1 stimulation [31, 32], we monitored the kinetics of p38 MAPK activation in PAR2 depleted PDAC cells by phospho-immunoblotting. Interestingly, levels of p-p38 MAPK were reduced after 1 h and 2 h of TGF-β1 stimulation in PAR2-depleted Panc-1 (Figure 4E) and IMIM-PC1 cells (data not shown), almost as much as after silencing of *TGFBRI* (Figure 4E). These data clearly show that the PAR2 effect on TGF-β signalling extends to non-canonical pathways as well.

Quantification of signal intensities by densitometry in Colo357 and IMIM-PC1 cells revealed that PAR2 depletion was generally not as potent as ALK5 depletion in decreasing the levels of p-Smad3C, p-Smad2C, and p-p38. However, combined depletion of PAR2 and ALK5 was not superior to ALK5 depletion alone in suppressing p-Smad3C (Figure 4B, 4C) and p-Smad2C (Supplementary Figure 3B, 3C), suggesting the possibility that PAR2 and ALK5 lie in the same pathway.

### Depletion of PAR2 decreases expression of ALK5

**Figure 5:**
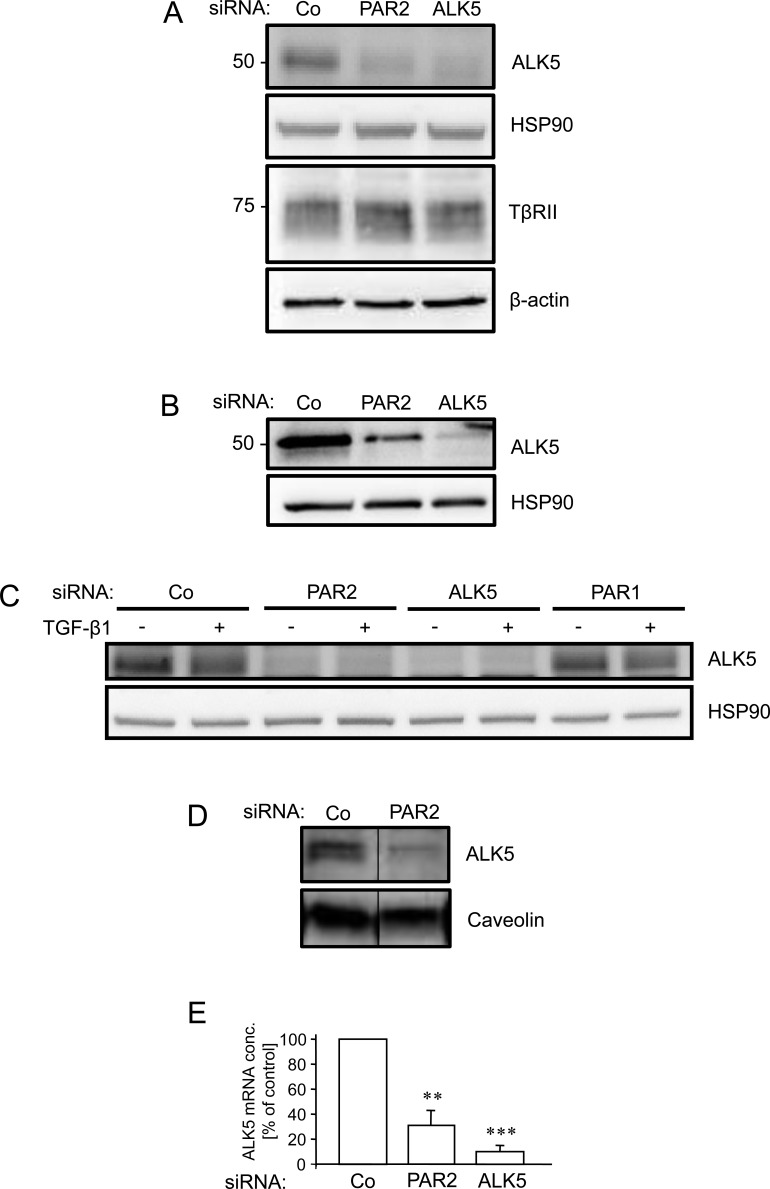
RNA interference mediated depletion of PAR2 suppresses expression of ALK5 Panc-1 **A.** Colo357 **B.**, and HaCaT **C.** cells were transfected twice on two consecutive days with 50 nM each of either control (Co) or PAR2 siRNA, and either ALK5 or PAR1 siRNA as positive and negative control, respectively. Forty-eight h after the second round of transfection, cells were lysed and the crude cellular lysates analysed by immunoblotting for expression of ALK5 and TβRII, and either HSP90 or β-actin as loading control. In C, HaCaT cells were treated, or not, with TGF-β1 for 1 h. **D.** As in A, except that preparations of membrane proteins were immunoblotted for ALK5 and the integral membrane protein caveolin as control. In each panel, representative blots are shown out of three (A-C) or two (D) experiments performed in total. Successful knockdown of PAR2, ALK5 and PAR1 expression was verified by qPCR (not shown). **E.** as in A except that cells were processed for RNA isolation and qPCR analysis of ALK5. Data are depicted as % inhibition relative to Co siRNA transfected cells (set at 100%, *n* = 5). Asterisks indicate significant differences relative to the control.

As shown above, depleting cells of PAR2 resulted in loss of Smad-dependent transcription (Figure 3) and Smad phosphorylation/activation (Figure 4). This result favors the idea that PAR2 promotes events upstream of Smad activation such as ligand binding, ligand-receptor complex formation, or receptor expression/function. Prompted by earlier studies showing that the ALK5 protein is targetted by cells to exert control over their sensitivity to TGF-β [35–37], we compared the abundance of ALK5 protein in control and PAR2 siRNA transfected cells. To this end, we noted a marked reduction in ALK5 protein levels in PAR2-depleted Panc-1 (Figure 5A), Colo357 (Figure 5B), and HaCaT cells (Figure 5C). In contrast, protein levels of TβRII were not affected by PAR2 depletion in Panc-1 cells (Figure 5A). To analyse whether the PAR2 knockdown-mediated decrease in total cellular ALK5 protein was also seen for the membrane associated fraction (which ultimately determines the sensitivity of cells to ligand), we measured the protein abundance of ALK5 in Panc-1 cell lysates enriched for membrane proteins. Again, ALK5 protein was strongly reduced in PAR2-depleted cells when compared with control cells (Figure 5D). Interestingly, we also observed downregulation of the ALK5 mRNA in Panc-1 cells (Figure 5E). Taken together, these data provide an explanation for the impaired sensitivity of PAR2 deficient PDAC cells to TGF-β1.

### Ectopic expression of wild-type and kinase-active ALK5 rescues TGF-β1-induced Smad phosphorylation, reporter gene activity and cell migration in PAR2 depleted cells

**Figure 6:**
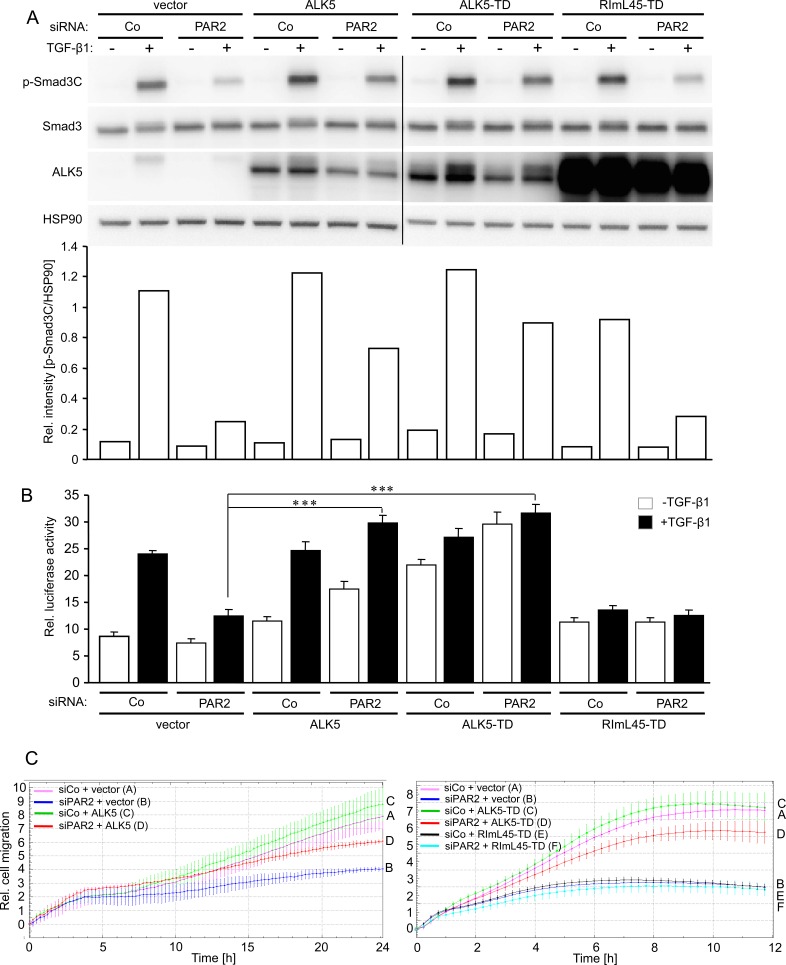
Ectopic expression of Smad binding competent ALK5 mutants can rescue TGF-β1-induced Smad activation, transcriptional activity, and cell migration **A.** Panc-1 cells were transiently transfected twice (on days 1 and 2) with 50 nM of either control siRNA (Co) or PAR2 siRNA and on day 2 additionally with either empty vector (vector), wild-type ALK5 (ALK5), kinase-active ALK5-T204D (ALK5-TD), or kinase-active ALK5 unable to bind Smads, RImL45-T204D (RImL45-TD). Forty-eight h after the second round of transfection, cells were stimulated with TGF-β1 for 1 h, lysed and analysed by immunoblotting for the indicated proteins. **A** densitometric analysis of underexposed replicas is shown below the immunoblots. Successful knockdown of PAR2, ALK5 and PAR1 expression was verified by qPCR (not shown). Note that RImL45-TD transfection in PAR2 depleted cells failed to increase p-Smad3C levels following TGF-β1 stimulation when compared to the respective vector control despite dramatic overexpression of RImL45-TD. **B.** As in **A**., except that cells received, in addition, p6SBE-Luc and pRL-TK-Luc and were stimulated with TGF-β1 for 24 h before they were subjected to dual luciferase assay. Data represent the normalised mean ± SD of six wells. The assay shown is representative of four experiments. **C.** Real-time cell migration assay of Panc-1 cells transiently transfected with control siRNA (siCo) or PAR2 siRNA (siPAR2), in combination with either empty vector or wild-type ALK5 (left-hand graph), or empty vector, ALK5-TD or RImL45-TD (right-hand graph). For the sake of clarity, only the data from TGF-β1-treated cells are shown. Significant differences between the siPAR2 + vector group (blue curves/B in both graphs) and the siPAR2 + ALK5 (red curve/D, left-hand graph) or the siPAR2 + ALK5-TD group (red curve/D, right-hand graph) were seen at 5:00 and 2:45, respectively, and all later time points. One representative assay out of three assays performed in total is shown. Letters to the right-hand side of the graph allow for a colour-independent identification of the various curves.

To analyse if the decrease in ALK5 abundance upon PAR2 depletion (see Figure 5) accounted for reduced Smad signalling and loss of TGF-β sensitivity, we attempted to rescue cells by transient ectopic expression of ALK5 using either the wild-type form, a kinase-active mutant, ALK5-T204D [38], or a Smad binding-defective mutant thereof, RImL45-T204D [39]. Notably, both wild-type ALK5 and ALK5-T204D, but not RImL45-T204D were able to partially restore p-Smad3C levels in TGF-β1-treated PAR2-depleted cells as compared to TGF-β1-treated cells that were transfected with control siRNA together with a control empty vector (Figure 6A). Next, we performed reporter gene assays to reveal whether supplying PAR2 depleted cells with ectopic ALK5 expression can overcome the loss of TGF-β /Smad transcriptional activity. Results showed that unlike RImL45-T204D, both wild-type and kinase-active ALK5 were able to rescue TGF-β1-induced transcriptional activity from p6SBE-Luc in the absence of PAR2 expression (Figure 6B). Moreover, both wild-type ALK5 (Figure 6C, left-hand panel) and ALK5-T204D (Figure 6C, right-hand panel), but not RImL45-T204D (Figure 6C, right-hand panel) were able to restore migratory activity in PAR2 depleted cells. Since overexpression of ectopic ALK5, as demonstrated exemplarily for the wild-type protein, was also seen in the membrane fraction of transfected cells (Supplementary Figure 4), we conclude that both wild-type ALK5 and its kinase-active mutant can rescue TGF-β /Smad3 signalling, TGF-β /Smad-dependent transcriptional activity and cell motility. Overall, these data suggest that PAR2 controls TGF-β signalling and cellular sensitivity through sustaining ALK5 protein expression.

## DISCUSSION

Both PAR2 and TGF-β share several functions in normal and pathological cellular physiology. With respect to cancer biology, both can drive tumour progression by promoting desmoplasia, migration, invasion, and metastasis [4–7,19–23]. Recently, we have provided evidence for a functional cooperation between PAR2 and TGF-β signalling in PAR2-AP-induced activation of Smad2 and upregulation of CTGF in human proximal tubular epithelial cells [26]. However, a role for PAR2 in TGF-β /ALK5 signalling in tumour cells was not known at that time. Prompted by the initital and unexpected observation that silencing of *F2RL1* by RNA interference in PDAC-derived cells abrogated TGF-β1-dependent Smad2/3C phosphorylation, we pursued the hypothesis that PAR2 was required for TGF-β1-dependent cellular responses relevant for tumour progression such as migration/invasion and invasion associated gene expression.

Using real-time cell analysis, we demonstrated that PAR2 expression was mandatory for TGF-β1-induced cell motility as evidenced by the observation that both the cells' migratory and invasive abilities were impaired after *F2RL1* silencing. Strikingly, the downregulation of PAR2 protein also prevented TGF-β1 from inducing the expression of proteinases (MMP2, MMP9) and proteinase regulators (PAI-1), thus providing a molecular correlate for the reduced invasive capacity of PAR2 deficient cells.

Since the control of both cell motility and growth arrest by TGF-β1 depends on a complex transcriptional program, we studied more directly whether PAR2 depletion in PDAC cells would affect general Smad dependent transcriptional activity. To this end, silencing of *F2RL1* dramatically reduced the activation of TGF-β /Smad responsive reporter genes, while ectopic expression of PAR2 enhanced this activity. Moreover, PAR2 depletion strongly decreased TGF-β1-induced Smad3 and Smad2 activation, and led to a failure of TGF-β1 to stimulate non-canonical *e.g*. p38 MAPK signalling (see Figure 4E) and transcriptional induction of its upstream activator GADD45β [31, 32] (see Figure 2B).

To assess whether PAR2 may have a more general role in TGF-β signalling that extends to non-human, non-epithelial and non-transformed cells, and whether this interaction may operate *ex vivo*, we tested the PAI-1 gene response to TGF-β1 in short-term cultures of smooth muscle cells derived from the aorta of PAR2^−/−^ and wild-type mice. Interestingly, primary aortic smooth muscle cells from the PAR2-null mice responded to TGF-β1 stimulation with a greatly attenuated induction of PAI-1 mRNA when compared to their wild-type counterparts.

A consistent observation was that the combined silencing of *F2RL1* and *TGFBRI* had no additive or synergistic effect on TGF-β1-induced Smad2/3 activation over that of silencing *TGFBRI* alone providing indirect evidence that PAR2 and ALK5 act together as a functional unit and in the same pathway during tumour progression as has recently been suggested for PAR2 and PAR1 [40]. Moreover, the finding that PAR2 interfered with Smad2/3C and p38 MAPK phosphorylation as well as transcriptional activation of TGF-β /Smad reporter and natural target genes indicated to us that PAR2 acted at the receptor level or upstream thereof. A clue regarding the mechanism came from the observation that siRNA mediated silencing of *F2RL1* resulted in reduced abundance of ALK5 protein, suggesting the possibility that the decline in ALK5 expression was responsible for the loss of TGF-β sensitivity. We confirmed this assumption by demonstrating that transfection of either wild-type ALK5 or a kinase-active and Smad binding-competent mutant of ALK5, but not a Smad-binding-defective derivative, was capable of rescuing PAR2 deficient cells from becoming refractory to TGF-β1-stimulated Smad activation, Smad transcriptional activity, and cell migration *in vitro*. In the ALK5 complementation experiments, we consistently observed lower protein levels of ectopically expressed ALK5 in PAR2 depleted *vs*. control cells (see Figure 6A, Supplementary Figure 4). This result was likely due to reduced DNA uptake and hence expression caused by arrested proliferation as a consequence of *F2RL1* silencing [15, 28]. Assuming a dose-dependency, a complete restoration of ALK5 kinase activity/Smad3C phosphorylation can be anticipated if equal levels of ectopic ALK5 expression in control and PAR2 siRNA transfected cells would have been achieved.

**Figure 7:**
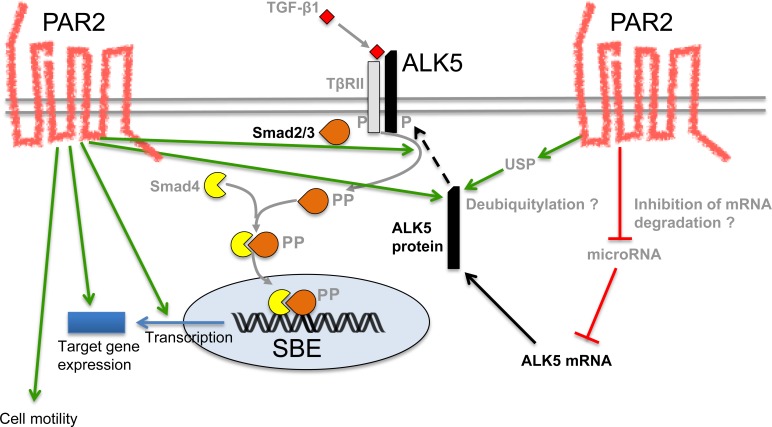
Schematic model illustrating potential mechanisms through which PAR2 regulates ALK5 expression and TGF-β signalling On the left-hand side, the green arrows indicate the various effects of PAR2 on the TGF-β pathway such as an increase in ALK5 protein abundance, R-Smad phosphorylation, Smad-mediated transcription, target gene expression and cell migration/invasion. On the right-hand side, still hypothetical mechanisms are shown that can lead to an increase in ALK5 protein such as deubiquitylation (eventually resulting in less degradation) or inhibition of a microRNA that targets ALK5 for degradation. The green arrows indicate stimulatory interactions and the red lines inhibitory interactions. The black stippled arrow indicates anterograde transport of ALK5 protein to the cell surface. P, phosphate residue; SBE, Smad binding element; USP, ubiquitin-specific protease.

Our current efforts are directed towards an elucidation of the molecular mechanisms involved in PAR2 control of ALK5 expression. In principal, this may occur at either the mRNA or protein level. For instance, deubiquitylation by the deubiquitylating enzymes ubiquitin-specific protease (USP)4, -11, or -15 has been shown to stabilise ALK5 protein through inhibition of proteosomal degradation [35–37]. It is thus conceivable that PAR2 acts as an inducer of USP expression and/or catalytic activity (Figure 7). Alternatively, given the decrease in ALK5 mRNA levels in Panc-1 cells in response to PAR2 depletion, PAR2 may act *via* an increase in *de novo* transcription of *TGFBRI* or *via* suppression of a microRNA that targets ALK5 mRNA for degradation (Figure 7). Besides mechanistic aspects of ALK5 regulation, we are attempting to identify the domains in PAR2 and the signalling pathways, if any, required for sustaining ALK5 expression.

Taken together, the results of our study indicate that in both tumourigenic PDAC and non-tumourigenic HaCaT cells and ASMCs, pro-migratory cellular responses to TGF-β1 require PAR2 protein expression. This may also extend to other gastrointestinal tumour types such as hepatocellular carcinoma as we could duplicate our findings in the hepatocellular carcinoma derived cell line Hep3B with stable expression of a PAR2 small hairpin RNA (H.U. and R.K., unpublished data). Our data strongly imply that PAR2, in addition to promoting migration and metastasis on its own [19–23], can enhance pro-oncogenic functions of TGF-β such as migration/invasion, and possibly metastasis, through its ability to promote ALK5 expression. We have recently obtained preliminary evidence that PAR2 is also mandatory for TGF-β1-induced EMT and EMT associated processes, such as cancer stem cell formation and acquisition of a chemoresistant phenotype. Moreover, through activating PAR2, not only trypsin but also FXa may be able to enhance TGF-β signalling. If so, this provides a potential pathophysiological mechanism linking pancreatic cancer to venous thromboembolism [41]. Hence, blocking PAR2 expression or function in the tumour microenvironment of advanced PDAC may be a promising novel approach to interfere with TGF-β 's pro-tumourigenic functions. Our observation of reduced induction by TGF-β1 of PAI-1 in primary ASMCs from PAR2 deficient mice may indicate that the PAR2-TGF-β signalling crosstalk is also operating *in vivo* and that it has pathophysiologic relevance for the development of vascular thrombotic and fibroproliferative disorders.

## MATERIALS AND METHODS

### Antibodies and reagents

The following antibodies were used: ALK5 (V-22), HSP90α/β (H-114, sc-7947), and PAR2 (SAM11, sc-13504, Fluorescein isothiocyanate (FITC)-conjugated) (Santa Cruz Biotechnology, Heidelberg, Germany), Caveolin (BD Transduction Laboratories, Heidelberg, Germany, #610059), phospho-p38 MAPK (Thr180/Tyr182), p38 MAPK, phospho-Smad2(Ser465/467) (all from Cell Signaling Technology, Frankfurt, Germany, #4370, #3104 and #2276, respectively), phospho-Smad3(Ser423/425) (R&D Systems, Wiesbaden, Germany, #ab3226), Smad2 (Epitomics, Burlingame, CA, #1736-1), Smad3 (Abcam, Cambridge, UK, #ab40854), TGF-β Receptor II (Cell Signaling Technology, #11888), β-actin (Sigma, Deisenhofen, Germany). TGF-β1 was purchased from either R&D Systems or ReliaTech (Wolfenbüttel, Germany) and SB431542 [42] from Sigma. Pharmacological inhibitors were added to cells 30-60 min before the addition of TGF-β1 which was used at a concentration of 5 ng/ml.

### Cells and animals

The TGF-β sensitive human PDAC cell lines Panc-1 and Colo357 were originally purchased from the ATCC and maintained as described earlier [43]. Another PDAC cell line, IMIM-PC1, was originally obtained from Dr. P. Real (University of Madrid, Spain) and kindly supplied by Dr. A. Menke (University of Giessen, Germany). HaCaT and HEK293T cells (both originally obtained from the ATCC) were kept in Dulbecco's modified Eagle's medium (DMEM) supplemented with 10% FCS, 1% glutamine and 1% penicillin/streptomycin. Prior to the experiments, all five cell lines underwent a short tandem repeat (STR)-based authentication by the Institute for Legal Medicine at the University Hospital Schleswig-Holstein. The results of the STR matching analysis (http://www.dsmz.de/fp/cgi-bin/str.html) confirmed the identity of the respective cell lines. All cells used in this study were routinely tested for the absence of mycoplasma contamination using the MycoAlert Mycoplasma Detection Kit (Lonza). Wild-type C57Bl/6 mice constitutively and globally deficient in PAR2 (PAR2^−/−^) on a C57Bl/6 background were housed at the animal research facility of the University of Greifswald on a standard laboratory diet (Ssniff^®^, Soest, Germany). The generation and characterisation of these mice was described earlier [44] and the knockout phenotype was recently confirmed by us using PCR-based genotyping (data not shown). These mice had successfully been employed in a number of studies from other investigators [45–47].

### Isolation, culture and TGF-β stimulation of primary mouse arterial smooth muscle cells

Mouse arterial smooth muscle cells from the thoracic aorta of wild-type and PAR2^−/−^ animals were prepared by enzymatic digestion according to the protocol described by Ross & Kariya [48]. Cells were used in passages 3-6 and cultured in DMEM (high glucose) supplemented with 10% fetal bovine serum (FBS, Life Technologies, Darmstadt, Germany), 200 U/ml penicillin, and 0.2 mg/ml streptomycin. For the experiments, cells were seeded into 6-well plates. At about 70% confluence, cells were incubated in serum-free DMEM for 24 h prior to incubation with TGF-β1.

### RNA isolation and qPCR analysis

Total RNA was isolated with PeqGoldRNApure (peqlab, Erlangen, Germany) and reverse-transcribed using Superscript II reverse transcriptase (Life Technologies). The qPCR reactions, the conditions of which were described in detail earlier [7], were performed on an I-cycler with IQ software (Bio-Rad, München, Germany). All values for the genes of interest were normalised to the housekeeping genes β-actin and TATA box-binding protein (TBP) for human genes and to glycerinaldehyde-3-phosphat-dehydrogenase (GAPDH) for murine genes. The sequences of PCR primers employed in qPCR are listed in Supplementary Table 1.

### Transient transfections and reporter gene assays

The siRNAs to PAR2, PAR1 and ALK5 (in each case a set of three prevalidated Stealth siRNAs, Validated Stealth RNAi^TM^ siRNA, Invitrogen). were used in combination, or singly for confirmation purposes, along with a Stealth siRNA negative universal control (for sequence information see Supplementary Table 2). The siRNAs were transfected twice (on two consecutive days) serum-free with Lipofectamine RNAiMAX (Life Technologies) according to the manufacturer‘s instructions. Transfected cells were subjected to immunoblot analyses, reporter gene assays, or cell migration assays. For the latter assays, cells were seeded in 96-well plates and were cotransfected on the next day serum-free with various siRNAs and Lipofectamine RNAiMAX. Twenty-four h reporter gene cells received Lipofectamine 2000 (Life Technologies) with the same siRNAs together with either p6SBE-Luc, p(CAGA)_12_ MLP-Luc or p3TP-Lux and the *Renilla* luciferase encoding vector pRL-TK-Luc. In some assays, an expression vector for PAR2-HA, ALK5-HA, ALK5-T204D-HA (a kinase-active mutant that in order to signal does not require ligand binding, or complex formation with TβRII [38], or RImL45-T204D-FLAG (a Smad-binding defective derivative of ALK5-T204D) [39] was cotransfected (see Supplementary Table 3 for a list of plasmid vectors and their suppliers). Each well received the same total amount of DNA. Forty-eight h after the start of the second transfection, cells were treated with TGF-β1 for 24 h and luciferase activities were determined with the Dual Luciferase Assay System (Promega). In all reporter gene assays, the data were derived from six wells processed in parallel and normalised with *Renilla* luciferase activity.

### Cell-electrode impedance migration and invasion assay

Using the xCELLigence^®^ DP device, we performed impedance based real-time measurements of random cell migration and invasion (chemokinesis) on non-transfected or transfected Panc-1, Colo357, and HaCaT cells. The migration assays were performed as decribed previously by us and others [49–51]. Each condition was performed in triplicate or quadruplicate with a programmed signal detection (RTCA software version 1.2.1., OLS, Bremen, Germany) every 15 min for a total of 12-48 h, depending on cell type and setup mode (migration or invasion). The setup of the invasion assay was identical to that of the migration assay except that the bottom of the wells was covered with a thin layer of Matrigel (5% (v/v), growth factor-reduced, BD Biosciences, Heidelberg, Germany, diluted 1:20 with basal medium) before cell seeding [49]. TGF-β1 was added to both lower and upper wells at the same concentration. In those experiments in which cells underwent transfection, they were processed to enter the assay 24-48 h after the second round of transfection.

### Immunoblot analysis of total and membrane proteins

Total cellular lysates were prepared in RIPA buffer (50 mM Tris, pH 8, 150 mM NaCl, 0.1% SDS, 0.5% sodium deoxycholate, 1% NP-40) containing protease inhibitor cocktail (Boehringer Complete) and phosphatase inhibitors (PMSF, NaF, sodium orthovanadate). For the isolation of membrane proteins cells were lysed in RIPA buffer and cleared from cellular debris in an Eppendorf centrifuge (2000 rpm, 10 min, 4°C). Lysates were subjected to ultracentrifugation (38 000 x g, 45 min). The resulting supernatant (the cytosolic fraction) was removed quantitatively and the pellet containing the detergent-resistant membrane fraction solubilised in lysis buffer containing 60 mM *n*-octyl β-D-thioglucopyranosidefor 1 h at 4°C. Following determination of the samples' protein concentrations with the Bradford assay (Bio-Rad), SDS-PAGE, transfer of fractionated proteins to PVDF membrane, and immunoblot analysis was performed as described previously [43].

### Statistical analysis

Student's *t*-test (unpaired, two-tailed) was used to compare two groups of independent samples to determine the probability of difference. Data were considered significant at *p* < 0.05. The *p*-values are indicated by asterisks, *, *p* < 0.05; **, *p* < 0.01; ***, *p* < 0.001.

## SUPPLEMENTARY MATERIALS FIGURES AND TABLES



## Abbreviations

ALK5: activin receptor-like kinase 5
ASMCs: arterial smooth muscle cells
FXa: activated factor X
EMT: epithelial-to-mesenchymal transition
FBS: fetal bovine serum
GAPDH: glycerinaldehyde-3-phosphat-dehydrogenase
MAPK: mitogen-activated protein kinase
MMP: matrix metalloproteinase
PAI-1: plasminogen activator inhibitor-1
PAR2: proteinase-activated receptor 2
PDAC: pancreatic ductal adenocarcinoma
qPCR: quantitative real-time RT-PCR
siRNA: small interfering RNA
TBP: TATA-box-binding protein
TGF-β: transforming growth factor-β
USP: ubiquitin-specific protease
