# Supplementary material for: Proteinase-activated receptor 2 promotes TGF-β-dependent cell motility in pancreatic cancer cells by sustaining expression of the TGF-β type I receptor ALK5
Source: Oncotarget. 2016 May 27;7(27):41095–109. doi: 10.18632/oncotarget.9600 (PMC5173045; doi:10.18632/oncotarget.9600)
Supplement: Supplementary file 1 [file oncotarget-07-41095-s001.pdf]

Proteinase-activated receptor 2 promotes TGF-β-dependent cell motility in pancreatic cancer cells by sustaining expression of the TGF-β type I receptor ALK5

Supplementary Material

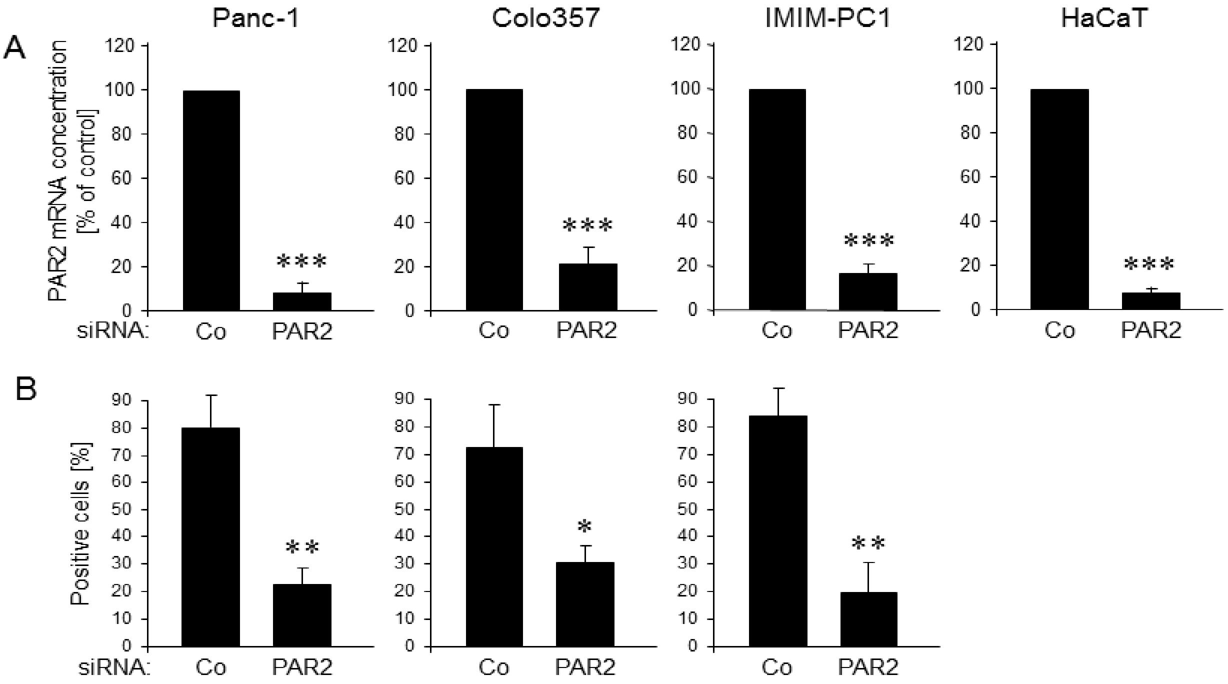

Supplementary Figure 1: Verification of successful siRNA-mediated silencing of *F2RL1* in the indicated cell lines. A, qPCR detection of PAR2 mRNA in PAR2 siRNA-transfected cells. Cells were transiently transfected twice with Lipofectamine RNAiMAX and either scrambled negative control siRNA (Co) or a mix of three specific PAR2 siRNAs (PAR2). Forty-eight h after the first transfection, cells were subjected to RNA isolation and qPCR analysis with PAR2-specific primers. Data (mean ± SD from 3 parallel wells were plotted in relation to those for controls set arbitrarily at 100%. B, Flow cytometry determination of surface-associated PAR2 protein following siRNA-mediated silencing of *F2RL1*. Cells were transfected as described in A and 48 h after the first transfection were subjected to flow cytometry analysis with PAR2-specific antibody as outlined in Materials and Methods. Data represent the mean ± SD , n = 3. *, $p < 0.05$; **, $p < 0.01$; ***, $p < 0.001$, unpaired, two-tailed Student's *t*-test.

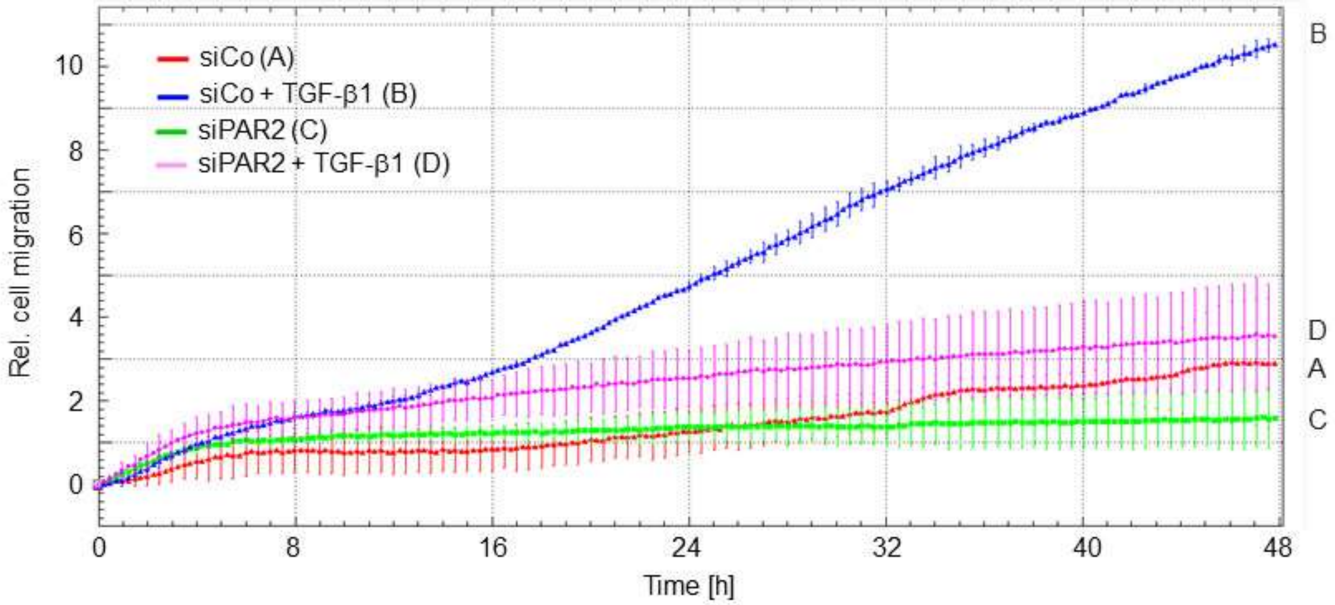

Supplementary Figure 2: SiRNA-mediated depletion of PAR2 suppresses TGF-β1-induced cell migration in HaCaT cells. Real-time measurement of cell migration in HaCaT cells transiently transfected with scrambled control siRNA (siCo) or PAR2 siRNA (siPAR2) and treated or not with TGF-β1. Data were derived from 3 parallel wells and represent the mean ± SD. One representative assay of 2 assays performed in total is shown. Data were significant between siCo+TGF-β1 (blue curve/B) and siPAR2+TGF-β1 (magenta curve/D) at 20:00 and all later time points. Letters on the graph's right-hand side allow for colour-independent identification of the curves.

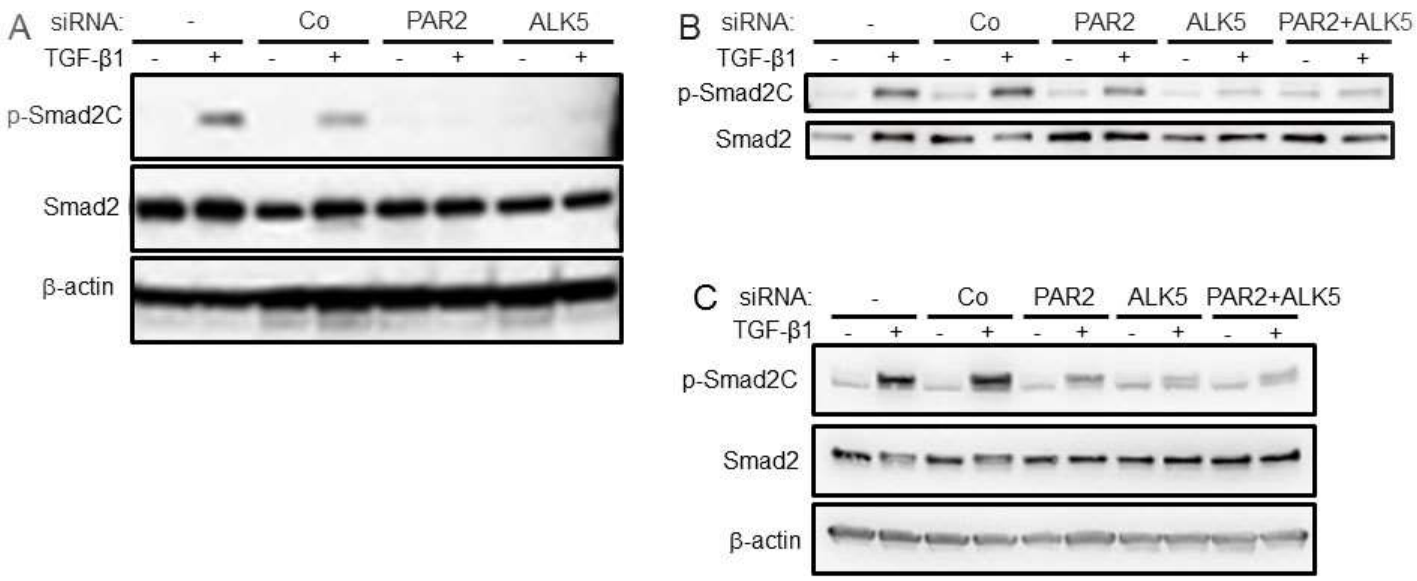

Supplementary Figure 3: Effect of *F2RL1* silencing on TGF-β1-induced phosphorylation of Smad2C in PDAC-derived cell lines. Panc-1 (A), Colo357 (B), and IMIM-PC1 (C) cells were transiently transfected twice with Lipofectamine (LFA) RNAiMAX alone (-) or LFA with 50 nM of either scrambled negative control siRNA (Co), a mix of three specific siRNAs for PAR2, ALK5 or PAR2+ALK5 as indicated. Forty-eight h after the first transfection, cells were subjected to a 1-h treatment with 5 ng/ml TGF-β1 followed by immunoblotting for p-Smad2C, and Smad2 and β-actin as loading control. Representative immunoblots from two independent experiments are shown.

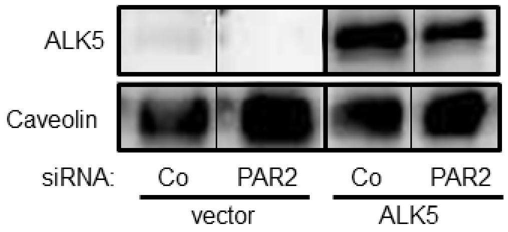

Supplementary Figure 4: Ectopically expressed ALK5 is cell membrane-associated. Panc-1 cells were transfected twice on two consecutive days with 50 nM of control (Co) or PAR2 siRNA, as indicated, using Lipofectamine 2000. For the second transfection, the transfection mix was supplemented with either an expression vector encoding wild-type ALK5 (ALK5) or empty vector (vector) as control. Forty-eight h after the second round of transfection, cells were lysed and subjected to membrane fractionation protocol as outlined in Material and Methods and subsequently analysed by immunoblotting for ALK5 and the integral membrane protein caveolin. One representative experiment out of three experiments performed in total is shown. Data are from the same blot and irrelevant lanes have been removed (indicated by the thin lines).

Supplementary Table 1: Primers used for qPCR

Primer name	Sequence (5'→3')	Expected size (base pairs)	GenBank accession
ALK5-forward	GCGACGGCGTTACAGTGTTTCTGC	378	NM_004612
ALK5-reverse	ATGGTGAATGACAGTGCGGTTGTGG	378	NM_004612
β-actin-forward	GACGAGGCCCAGAGCAAGAG	785	NM_001101
β-actin-reverse	ATCTCCTTCTGCATCCTGTC	785	NM_001101
GADD45β-forward	AACATGACGCTGGAAGAGCTCG	490	NM_015675
GADD45β-reverse	CTCAGCGTTCCTGAAGAGAGATG	490	NM_015675
GAPDH-forward	TTGCCATCAATGACCCCTTCA	174	NM_001289745.1
GAPDH-reverse	CGCCCCACTTGATTTTGGA	174	NM_001289745.1
mGAPDH	Mm99999915_g1 Gapdh FAM, TaqMan Gene Expression Assay (ABI / ThermoFisher Scientific)		NM_008084.3
MMP2-forward	CACCCTGGAGCGAGGGTAC	465	NM_004530.5
MMP2-reverse	CTGATTAGCTGTAGAGCTGAAGGC	465	NM_004530.5
MMP9-forward	CATTTCGACGATGACGAGTTGT	230	NM_004994
MMP9-reverse	CGGGTGTAGAGTCTCTCGC	230	NM_004994
PAI-1-forward	CTTCTTCAGGCTGTTCCGGAGC	808	X04744
PAI-1-reverse	GGGTCAGGGTTCCATCACTTGG	808	X04744
mPAI-1	Mm00435858_m1 Serbp1 FAM, TaqMan Gene Expression Assay (ABI / ThermoFisher Scientific)		NM_008871.2
Smad7-forward	GGAAGATCAACCCCGAGCTG	200	NM_005904
Smad7-reverse	TTGGGAATCTGAAAGCCCCC	200	NM_005904
PAR1-forward	TGTGTACACCGGAGTGTTTGTAG	264	NM_001992
PAR1-reverse	ACTGTCATGAGCAAGATAGAGGC	264	NM_001992
PAR2-forward	ACTCCAGGAAGAAGGCAAACA	98	NM_005242
PAR2-reverse	TGGTCTGCTTCACGACATACA	98	NM_005242
TBP-forward	GCTGGCCCATAGTGATCTTT	60	M55654.1
TBP-reverse	CTTCACACGCCAAGAAACAG	60	M55654.1

Supplementary Table 2: SiRNAs used for cell transfection

SiRNA	Supplier	Cat. #	Sequence	GenBank accession
ALK5	Invitrogen	HSS110695<br>HSS110696<br>HSS110697	GCAUCUCACUCAUGUUGAUGGUCUA<br>GCCAAAUGAAGAGGACCCUUCAUUA<br>GGAGAAGAAGUUGCUGUUAAGAUAU	NM_004612
PAR1	Invitrogen	HSS103468<br>HSS176655<br>HSS176656	GCUGAUCAUUUCCACGGUCUGUUAU<br>GAAUACAGAUUAGUCUCCAUCAAUA<br>UGUUUGUAGUCAGCCUCCCACUAAA	NM_001992
PAR2	Invitrogen	HSS103471<br>HSS103472<br>HSS103473	UCAACCACUGUUAAGACCUCCUAUU<br>GGGAAGCUCUUUGUAAUGUGCUUAU<br>GCAACAUGUACUGUUCCAUUCUCUU	NM_005242
Stealth^{TM} RNAi Negative Control	Invitrogen	12935-200		

Supplementary Table 3: Reporter genes and expression plasmids used for cell transfection

Plasmid	Supplier	Cat. #	Vector (Supplier)	Ref./GenBank accession
p6SBE-luc	S. E. Kern, Baltimore, MD		pGL3-Promoter (Promega)	Ref. 1[a]
p(CAGA)$_{12}$ MLP-Luc	S. Dooley, Mannheim, Germany		pGL2 (Promega)	Ref. 2[b]
p3TP-Lux	J. Massaguė, NY		pGL2-Basic (Promega)	Ref. 3[c]
pRL-TK-Luc	Promega, Heidelberg, Germany	E2241	pRL *Renilla* Luciferase Reporter Vector	
PAR2-HA	S. Compton, Cottingham, UK		pcDNA3.1(-)	NM_005242
ALK5-HA	MRC-PPU Reagents, University of Dundee, Scotland	DU33064	pCMV5	NM_004612
ALK5-T204D-HA	J. Massaguė, NY		pCMV5	NM_004612
RImL45-T204D-FLAG	Y. E. Zhang, Bethesda, MD		pCMV5	NM_004612

[a]Ref. 1: Zawel L, Dai JL, Buckhaults P, Zhou S, Kinzler KW, Vogelstein B, Kern SE. Human Smad3 and Smad4 are sequence-specific transcription activators. Mol Cell. 1998; 1:611-617.

[b]Ref. 2: Dennler S, Itoh S, Vivien D, ten Dijke P, Huet S, Gauthier JM. Direct binding of Smad3 and Smad4 to critical TGF beta-inducible elements in the promoter of human plasminogen activator inhibitor-type 1 gene. EMBO J. 1998; 17:3091-3100.

[c]Ref. 3: Wrana JL, Attisano L, Cárcamo J, Zentella A, Doody J, Laiho M, Wang XF, Massagué J. TGF beta signals through a heteromeric protein kinase receptor complex. Cell. 1992; 71:1003-1014.
